# Postpolypectomy Electrocoagulation Syndrome: A Mimicker of Colonic Perforation

**DOI:** 10.1155/2013/687931

**Published:** 2013-07-15

**Authors:** Brian C. Benson, Jonathan J. Myers, Jeffrey T. Laczek

**Affiliations:** ^1^Department of Medicine, Tripler Army Medical Center, 1 Jarrett White Road, Honolulu, HI 96859, USA; ^2^Gastroenterology Service, Tripler Army Medical Center, USA

## Abstract

Postpolypectomy electrocoagulation syndrome is a rare complication of polypectomy with electrocautery and is characterized by a transmural burn of the colon wall. Patients typically present within 12 hours after the procedure with symptoms mimicking colonic perforation. Presented is the case of a 56-year-old man who developed abdominal pain six hours after colonoscopy during which polypectomy was performed using snare cautery. CT imaging of the abdomen revealed circumferential thickening of the wall of the transverse colon without evidence of free air. The patient was treated conservatively as an outpatient and had resolution of his pain over the following four days. Recognition of the diagnosis and understanding of the treatment are important to avoid unnecessary exploratory laparotomy or hospitalization.

## 1. Introduction

Postpolypectomy electrocoagulation syndrome (also known as postpolypectomy syndrome or transmural burn syndrome) is characterized by peritoneal inflammation in the absence of frank perforation occurring after polypectomy with electrocautery. It is a rare complication of polypectomy and occurs when electrical current applied during polypectomy extends into the muscularis propria and serosa resulting in a transmural burn at the site of polypectomy. Patients typically present with abdominal pain and tenderness hours to days after the procedure but may also have fever, tachycardia, and leukocytosis; their presentation often mimics colonic perforation. Recognition of the diagnosis and understanding of the treatment is important to avoid unnecessary exploratory laparotomy or hospitalization.

## 2. Case Report

**Figure 1:**
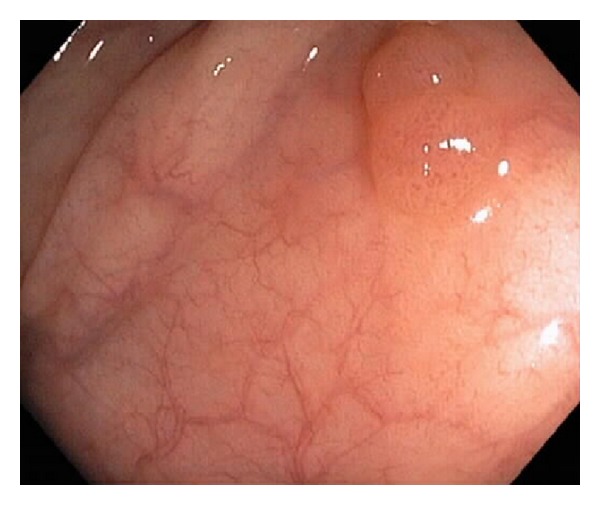
Tubular adenoma removed with snare cautery.

**Figure 2:**
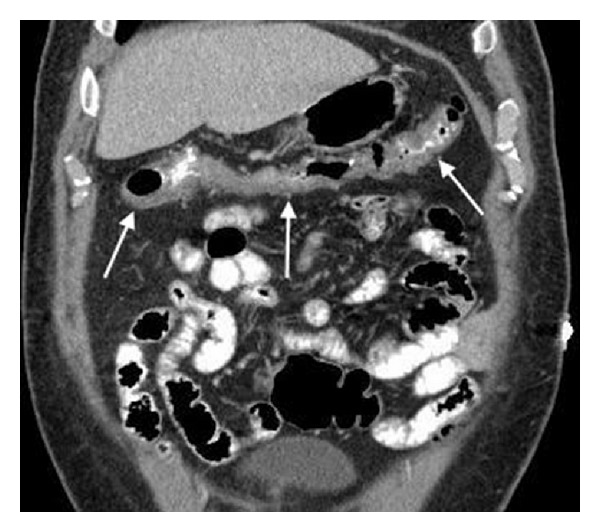
Long segment of circumferential wall thickening of transverse colon.

**Figure 3:**
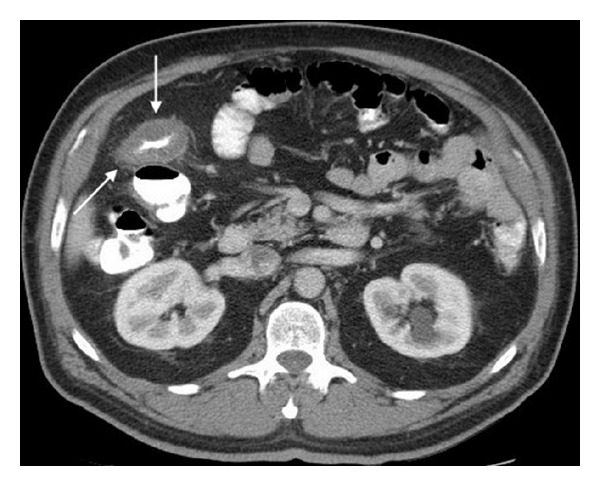
Circumferential wall thickening of transverse colon.

A 56-year-old man underwent a colonoscopy for initial colorectal cancer screening, revealing an 8 mm transverse colon polyp (Figure 1) which was removed with snare cautery and a 6 mm transverse colon polyp that was removed with a cold snare. The evening after the procedure, he presented to the emergency department complaining of acute onset of diffuse, sharp abdominal pain beginning approximately six hours after his colonoscopy. He reported one episode of nonbloody diarrhea shortly after his colonoscopy but had no symptoms of nausea or vomiting. He was found to be afebrile and had normal vital signs. Physical exam was notable for moderate right lower quadrant abdominal tenderness without peritoneal signs or rebound tenderness. Laboratory studies were significant for a leukocytosis of 18.0 × 10^9^/L with 87% granulocytes; chemistries and hepatic function studies were within normal limits. A plain film of the abdomen was normal. A CT scan of the abdomen with IV and oral contrast revealed a long segment of circumferential wall thickening extending from the mid ascending colon to the splenic flexure (Figures 2 and 3). General surgery and gastroenterology consults were obtained while the patient was in the emergency department. He was diagnosed with postpolypectomy electrocoagulation syndrome and treated conservatively without antibiotics. He had complete resolution of abdominal pain over the following four days.

## 3. Discussion

The postpolypectomy electrocoagulation syndrome is a rare complication of polypectomy occurring in 0.14% to 2% of patients who undergo polypectomy [1–3]. A large retrospective cohort identified this syndrome as a complication that occurred in only 6 of 16,318 colonoscopies [4]. The syndrome occurs when electrical current applied during polypectomy extends into the muscularis propria and serosa resulting in a transmural burn at the site of polypectomy. Patients typically present with symptoms of abdominal pain and tenderness within 12 hours after the polypectomy, although a presentation as late as five days after the procedure has also been reported [5]. These patients may also have fever, tachycardia, and leukocytosis which often mimics colonic perforation, a rarer but more serious complication occurring in 0.07% to 0.3% of patients who undergo polypectomy [5–7]. 

Abdominal radiographs and basic blood work to include a complete blood cell count are part of the routine evaluation in patients presenting with abdominal pain following a colonoscopy. Patients who underwent a polypectomy with electrocoagulation and who have concerning findings on evaluation such as fever, leukocytosis, or peritoneal signs but have no evidence of perforation on radiographs should be evaluated with further imaging such as a contrast enhanced CT scan. In patients with bowel wall thickening consistent with postpolypectomy electrocoagulation syndrome, the treatment includes supportive care to include observation, analgesia, IV fluids, bowel rest, and possibly antibiotics. The decision to treat using antibiotics is based on the overall clinical appearance of the patient. If prescribed, antibiotics should cover gram negative and anaerobic bacteria.

In conclusion, this case illustrates the potential for postpolypectomy syndrome to mimic more ominous complications of colonoscopy. Postpolypectomy syndrome should be considered in patients who develop abdominal pain, fever, leukocytosis, and/or signs of peritoneal inflammation following polypectomy with electrocautery. The distinction from colonic perforation requires radiologic evaluation which is best accomplished with abdominal CT scan. It should be treated conservatively with intravenous fluids, avoidance of oral intake, bed rest, and potentially antibiotics. Moderate cases, such as this one, can be managed without hospital admission.
